# Health care professionals’ perspectives on the utilisation of a remote surveillance and care tool for patients with COVID-19 in general practice: a qualitative study

**DOI:** 10.1186/s12875-022-01863-z

**Published:** 2022-09-27

**Authors:** Mariell Hoffmann, Sandra Stengel, Joachim Szecsenyi, Frank Peters-Klimm

**Affiliations:** Department of General Practice and Health Services Research, Heidelberg University Hospital, Im Neuenheimer Feld 130.3, 69120 Heidelberg, Germany

**Keywords:** COVID-19, Surveillance tool, Care tool, General practice, Process evaluation, Remote, Qualitative study, RE-AIM

## Abstract

**Background:**

Most COVID-19 patients with severe symptoms are treated in hospitals. General practices are responsible for assessing most ambulatory patients. However, they face several challenges managing COVID-19 patients, and those with non-COVID-19 conditions. In April of 2020, we designed a software tool for the structured surveillance of high-risk home-quarantined COVID-19 patients in general practice (CovidCare) including several telephone monitorings, in order to support general practices and early identification of severe courses. This study presents the qualitative results of a mixed-methods process evaluation study on CovidCare.

**Methods:**

In a qualitative process evaluation study conducted between March and May 2021, we explored the perspectives of seven general practitioners (GPs) and twelve VERAHs (medical care assistants with special training) on CovidCare using semi-structured interviews based on the RE-AIM framework (reach, effectiveness, adoption, implementation, maintenance). We used deductive qualitative content analysis employing the RE-AIM framework to assess the utilisation and implementation of CovidCare.

**Results:**

Overall, most health care professionals were satisfied with CovidCare. They highlighted 1) a good orientation for the management of COVID-19 patients, especially due to a high level of uncertainty at the beginning of the pandemic, 2) the possibility to gain new knowledge, and 3) the structured data collection as facilitators for the implementation of CovidCare. Moreover, CovidCare reduced the workload for GPs while some VERAHs perceived a higher workload as they were responsible for large parts of the CovidCare management. However, CovidCare positively affected the VERAHs’ job satisfaction as most patients provided positive feedback and felt less anxious about coping with their disease. Previous experience with the software and an easy integration into daily practice were considered to be crucial utilisation drivers. Time and personnel resources were identified as major barriers. To further improve CovidCare, participants suggested a less comprehensive version of CovidCare, the expansion of inclusion criteria as well as an app for the patients’ self-management.

**Conclusion:**

The COVID-19 surveillance and care tool for COVID-19 patients with increased risk was perceived as useful by GPs and VERAHs. Supportive remote health care tools such as CovidCare are a viable means to maintain comprehensive and continuous health care during a pandemic and may strengthen the primary care system.

**Trial registration:**

German Clinical Trials Register DRKS00022054; date of registration: 02/06/2020.

**Supplementary Information:**

The online version contains supplementary material available at 10.1186/s12875-022-01863-z.

## Background

The COVID-19 pandemic has created an enormous challenge to health care systems. While COVID-19 patients with severe symptoms are treated in hospitals, general practices are responsible for those who do not need to be hospitalised. These infectious patients must be managed in domestic isolation. In case of a deterioration, a timely identification allows an effective management. However, especially in the beginning of the pandemic, general practices had to deal with a lack of clear recommendations and therapeutic approaches as well as rapidly changing guidelines for the management of COVID-19 patients. Moreover, in order to prevent infection of and health care providers (HCP) and other patients, a rapid development of novel and dynamic ways of working became necessary [1, 2].

Telehealth approaches are primed to enable HCP to provide virtual care for COVID-19 patients and patients with regular health issues and can be an effective way of treatment [3–5]. There are many different models for the remote management of COVID-19 patients in long-term care facilities [6], hospitals [7] and practices [8], e.g. including symptom tracking by HCP or the patients themselves, remote monitorings and follow-ups. Besides, apps, e.g. to support HCP in collecting data on the health status of patients who are self-managing [9, 10] in home-quarantine, have also been developed [11–13] and can support HCP in the early detection of deterioration [6, 10]. Evaluations of these models points to several crucial perquisites for the remote management of COVID-19 patients such as the integration of the patients’ data into softwares used in general practices [7, 8] to reduce workload and foster implementation, as well as a close contact between the patients and the doctors especially for high risk patients with chronic diseases [8]. Furthermore, previous work highlight the use of a symptom diary as particularly useful for the management of COVID-19 patients [9]. These tools should also be evaluated for additional scientific value and to ensure quality standards [7]. However, most models to date only include contact to HCP from the patients’ initiative, which might impede an early detection of signs of deterioration and effective management of COVID-19 patients.

In Germany, around 160 000 GPs and specialists provide ambulatory care, mostly in private practice, and are reimbursed by statutory health insurances, primarily using fee-for-service payments. The majority of the German population is member of the statutory health insurance. Most general practitioners are self-employed and work together with medical assistants. Within the framework of selective contracts for general-practitioner-centered care (Hausarztzentrierte Versorgung), experienced medical assistants with additional training (Care Assistant in General Practice with special training, Versorgungsassistentin in der Hausarztpraxis, VERAH) support the GPs work. General practitioners work as gatekeepers for patients, assess their state of health and refer to a specialist if necessary. As most COVID-19 patients are treated by general practitioners, a comprehensive approach is necessary to support general practices in the management of COVID-19 patients, providing a structured way to detect deterioration, treat patients individually, and reducing uncertainty for both patients and HCP. Hence, we designed a COVID-19 surveillance and care tool for COVID-19 patients with increased risk (CovidCare) [14]. CovidCare aims to support general practices in providing comprehensive and continuous health care, aimed at a reduction of uncertainty of the COVID-19 patient and HCP likewise and indirectly the number of avoidable hospitalisations. As large parts can be conducted by the VERAH (the workload for general practitioners (GPs) shall be reduced through task delegation and shared responsibilities.

CovidCare is accompanied by a multi-center prospective longitudinal noncontrolled observational study exploring the effect of different risk factors on disease progression [15]. Furthermore, the process evaluation, designed as an exploratory sequential mixed-methods study, explores the utilisation of CovidCare from the perspective of participating HCP in order to make adaptions to CovidCare to improve the fit between CovidCare and the general practices from the users’ perspectives. In this study, we present the results from the qualitative strand of the process evaluation, based on interviews with GPs and VERAHs who used CovidCare in daily practice.

## Methods

### Overview

The CovidCare-module was built on top of an existing platform named CareCockpit which was developed and is licensed by the Department of General Practice and Health Services Research at the Heidelberg University Hospital, and was designed for case management in general practices. CovidCare aims to support general practice teams to take care of confirmed COVID-19 patients in a structured and semi-standardized way. It consists of three core components: 1) the patient intake (conducted by the VERAH or GP) which includes the collection of master data such as name, address and a check for inclusion criteria; 2) the assessment (GP and VERAH) in which information on risk factors for a severe course of the disease, living condition and symptom history is collected; patients can also be provided with a symptom diary that can be used for the daily log of symptoms by the patients themselves; and 3) several telephone monitorings (VERAH) which include the documentation of symptoms and signs of infection and deterioration. The final consultation (VERAH and GP) includes a short closing documentation of different health care outcomes such as death and health care utilisation (e.g. hospitalization).

All GPs who participate in general-practice centred care (GP-centred care) by the AOK Baden Württemberg (large German sickness fund) are eligible for using CovidCare. To be managed in CovidCare, patients have to be at least 18 years old, insured with the AOK Baden Wuerttemberg, participate in GP-based care, PCR-tested for SARS-CoV-2 and have at least one risk factor for a severe course of the disease according to the Robert-Koch Institute (e.g. > 50 years, diabetes, cardiovascular disease, pulmonary disease, obesity (BMI > 30) [16]. Overall, a total number of at least three to four contacts is recommended, including the intake, the initial assessment and a closing contact. For each patient meeting the inclusion criteria and managed with CovidCare, the general practice receives 40 Euro remuneration.

Against the background that COVID-19 related studies were scarce, we designed a multi-center prospective longitudinal non-controlled observational study in April 2021 during the first wave of the COVID-19 pandemic in Germany. A detailed description of the intervention and the CovidCare study is provided in our study protocol [15]. The accompanying process evaluation explored the utilisation of CovidCare from the perspectives of HCP and patients and follows an exploratory sequential mixed-methods design using the RE-AIM framework to guide data collection and data analysis [17, 18]. RE-AIM is an established framework to evaluate interventions and allows to comprehensively cover multiple aspects of a program. This is particularly necessary for our study as also aim to improve and further tailor CovidCare it to the HCP’s needs.

In a first step, qualitative data were collected and analysed. Second, questionnaires will then be developed based on the qualitative results to gain a comprehensive understanding of the utilisation of CovidCare. This article presents the qualitative findings from the first part of the process evaluation with the HCP during the third wave of the COVID-19 pandemic in Germany.

### Design and setting

We followed a naturalistic-qualitative inquiry using interviews with open-ended questions. Thus, we were able to account for a process orientation that explores perspectives on CovidCare under real-world conditions [19]. This also acknowledges the purpose of gaining insights into the day-by-day reality of the HCP who implemented CovidCare and the crucial suggestions for further improvements of CovidCare in order to facilitate the integration into HCP’s working routines.

We designed a cross-sectional qualitative study and collected data from interviews to evaluate the utilisation of CovidCare. We used the RE-AIM framework to guide our evaluation on reach, efficacy, adoption, implementation and maintenance of CovidCare [17]. RE-AIM was the appropriate framework because it is designed to evaluate real-world interventions and to guide data collection as well as data analysis. The study population consists of GPs and VERAHs who used CovidCare and participated in the CovidCare study.

### Recruitment and sampling

We applied purposeful sampling in order to select those participants who are experienced with CovidCare and have already used it to manage COVID-19 patients in their general practice and are willing to participate in an interview [20]. We invited all GPs and VERAHs who participated in the CovidCare study until 26/06/2021 to take part in an interview. Specifically, we sent an invitation letter and a response coupon with which they can declare their interest in interview participation using an enclosed postpaid envelope to 96 general practices, including 112 GPs and 105 VERAHs. We sent a written reminder to all non-responders after four to eight weeks. Overall, 32 GPs (35.8%) and 43 VERAHs (45.2%) responded to the interview invitation while 11 GPs and 16 VERAHs showed interest in participating in an interview. MH (female research fellow, sociologist, expertise in qualitative research) contacted all interested participants via telephone to provide more information on the interviews and schedule an interview appointment. Eight persons did not take part in an interview due to organizational reasons or because they have not yet used CovidCare which was a prerequisite for interview participation. In total, we conducted telephone interviews with seven GPs and 12 VERAHs from 15 different general practices (17–48 min). All VERAHs were offered a non-advertised individual monetary compensation of €30. GPs were not offered an individual monetary compensation.

### Data collection

Prior to the study, we developed a semi-structured interview guide in a multidisciplinary team (sociology, general practice) (see Additional file 1) based on the dimensions of the RE-AIM framework which was reviewed by the research team as well as after the first two interviews. According to RE-AIM, the questions focused on reach (e.g. willingness of HCP and patients to be reached by CovidCare), efficacy (e.g. positive and negative effects for patients and HCP), adoption (e.g. reasons for using CovidCare), implementation (e.g. problems and adaptions that were necessary) and maintenance (factors that facilitate the uptake of CovidCare) [17]. All telephone interviews were conducted by MH, who was not acquainted to the participants prior to the study, between March and May 2021. At the beginning of each interview, the purpose was stated as being to explore the HCP’s experiences and perspective on CovidCare. Data protection guidelines and study objectives were made transparent to all participants who all gave their written informed consent prior to data collection. All interviews were audio-recorded, digitalized and stored at the Department of General Practice and Health Services Research, Heidelberg University Hospital and were only accessible to the study team. We complemented the interviews with field notes. We did not repeat any interviews or returned transcripts to the interviewees for feedback on the findings. However, as part of the quantitative strand of the process evaluation, we will derive key messages from the qualitative data and evaluate them in the subsequent second part of the process evaluation. For reporting, we followed the COREQ guidelines [21] (see Additional file 2).

### Data analysis

Three research assistants of the department conducted verbatim transcriptions of all interviews. Identifiable information such as names and places were masked. For data analysis, two researchers carried out a deductive qualitative content analysis [19]. MH and SS (female general practitioner, expertise in qualitative research) independently re-read the same two transcripts and deductively applied the RE-AIM dimensions to the data as laid out in the study protocol [15] using MAXQDA 2018. Both coders compared their coded sections, discussed discrepancies (e.g. regarding the assignment to each dimension) and drafted code definitions. Subsequently, both coders independently re-read and coded another transcript and met again to resolve questions. MH analysed the remaining transcripts based on the RE-AIM dimensions and the code definitions and met with SS for constant comparison to ensure data consistency. Data saturation has been reached when a wide range of HCP who used CovidCare in a different manner (e.g. number of included patients, time of use) had been interviewed and all perspectives were represented in the coded sections [22]. An overview of the key dimensions and subdomains as well as code definitions and supporting quotes is provided in the appendix (Additional file 3).

### Ethics approval and consent to participate

This study is embedded in a mixed-methods process evaluation as part of the CovidCare study [15], which received ethical approval by the ethics committee of the Medical Faculty of the University of Heidelberg (reference number: S.266/2020). All participants signed the informed consent forms.

## Results

Prior to exploring the RE-AIM dimensions in detail, we assessed how the interviewees managed COVID-19 patients before using CovidCare. Results on the RE-AIM dimensions will subsequently be described including quotes from the interviews.

### Sample

Table 1 shows the sociodemographic characteristics of the seven GPs and 12 VERAHs interviewed.

**Table 1:** Participants’ characteristics

Specialty	n (%)	Gender Male n (%)	Gender female n (%)	500–1000 patients per quarter, n (%)	1000–1500 patients per quarter, n (%)	> 1500 patients per quarter, n (%)
GPs	7 (36.8)	2 (10.5)	5 (26.3)	3 (15.8)	0 (0)	4 (21.0)
VERAHs	12 (63.2)	0 (0)	12 (63.2)	3 (15.8)	1 (5.3)	3 (15.8)
**Total**	19 (100)	2 (10.5)	17 (89.5)	6 (31.6)	1 (5.3)	7 (36.8)

### Management of COVID-19 patients before using CovidCare

The interviewees presented heterogenous statements on how they managed COVID-19 patients before using CovidCare. While some described that COVID-19 patients were asked to call the general practice as soon as symptoms worsen, others also called the patients themselves to ask about their condition. Medication prescriptions were also used to relieve the most prominent symptoms. One GP reported that she developed a monitoring checklist herself to systematically collect symptoms and vital signs of COVID-19 patients. Overall, HCP reported that they were overwhelmed with the treatment of COVID-19 patients due to a high level of uncertainty caused by a lack of evidenced-based guidelines among other factors.

### Reach

Reach describes the extent to which patients are reached by the implementation of CovidCare and refers to (the number of) patients who were willing or refused to be managed with CovidCare and why.

According to the patients’ inclusion criteria for CovidCare, HCP reported that all participating patients were aged 18 years or older and most had at least one risk factor, most commonly diabetes and/or hypertension. The interviewees stated that they also included patients who did not meet (all) of the inclusion criteria and for whom they did not receive remuneration.

*“we use it for all patients of whom we think they benefit from it [being managed in CovidCare]“ (Participant 7, GP)*

Specifically, in order to provide adequate health care for all COVID-19 patients, HCP also managed patients in CovidCare who were not insured with the AOK Baden Württemberg and/or did not participate in GP-based care and/or did not have a risk factor for a severe course of COVID-19. Included patients had different symptoms such as *“unspecific symptoms” (Participant 9), “signs of cold and influenza” (Participant 9 and participant 13)*, strong cough and fever, ranging from mild to severe forms.

Furthermore, HCP perceived patients’ willingness to be managed in CovidCare as rather high, especially in older patients:

*“Well, I haven’t seen anyone who hesitated in any way.” (Participant 18, GP)*

Some interviewees mentioned that rather younger patients were more reluctant because “*younger patients perceive this [the COVID-19 disease] as normal cold” (Participant 1, VERAH)*.

### Effectiveness

Effectiveness is defined as positive and negative outcomes for patients and HCP, the patients’ quality of life as well as the patients and the HCP’s satisfaction with CovidCare.

Overall, the interviewees were largely satisfied with CovidCare. They regarded CovidCare as guidance for the management of COVID-19 patients and highlighted the possibility to collect data in a structured way. Hence, against the background of a scarce, changing and confusing body of official guidelines especially at the beginning of the pandemic, CovidCare provided a feeling of security and increased their confidence to adequately treat their patients.

*“[CovidCare] has really paved the way for us, simply knowing what to look out for (…) [that] has also given me a certain sense of security. Also, that these symptom diaries served as guideline (…) it really was a great support.” (Participant 16, VERAH)*

*“Simply a better overview, through this monitoring… also a good control that you don’t miss anything.” (Participant 5, VERAH)*

Moreover, some HCP contributed their satisfaction to the possibility to gain new knowledge about their individual patients and about the COVID-19 disease in general, adding that the recurring telephone calls and additional time spend for CovidCare helped them to *“learn things [about the patient] we otherwise would not have learnt” (Participant 4, GP).*

However, despite the overall satisfaction with CovidCare, few GPs perceived CovidCare as *“rather uneasy to handle” (Participant 16, GP)* with regard to the use of the software and the installation process and stated that CovidCare included too many questions.

Some statements other varied between GPs and VERAHs. More precisely, GPs valued a relief of their workload as particularly positive outcome of using CovidCare. Thus, they were able to delegate rather time-consuming telephone follow-up calls to the VERAH while still being able to keep track of their patients, e.g. detect severe courses at an early stage and respond appropriately in a timely manner when necessary:

*“For us, it actually worked out quite well, especially for us as doctors, because it relieves us a lot, to know that the VERAH simply calls the patients regularly and if anything happens, then (…) they give feedback. And then I can also call the patients again.” (Participant 7, GP)*

In contrast, VERAHs emphasized additional workload as particularly negative. Against the background of an already tightly organized daily practice, e.g. due to an increased number of telephone calls during the pandemic and the management of COVID-19 vaccinations, some VERAHs considered the *“additional work and additional time” (Participant 8, VERAH)* related to CovidCare to have put an additional strain on meeting the increased requirements of their day-to-day work. However, some VERAHs highlighted that CovidCare positively affected their job satisfaction adding that patients provided particularly positive feedback for conducting the recurring follow-up calls and spending extra time on the patient.

*“It just strengthened the VERAH in their work, so you just felt good and I have never gotten such positive recognition of my work as I have during this time.” (Participant 1, VERAH)*

As for patients, HCP underscored that patients were largely satisfied being managed with CovidCare. From the perspective of the HCP, patients felt well looked after, especially because many patients felt insecure due to their COVID-19 disease.

*“We gave them [patients] tasks like measuring their blood pressure and they were supposed to write everything down in the symptom diary and that always worked out great. They were motivated and (…) most of them [had] everything ready, had written everything [all vital signs] down.” (Participant 17, VERAH)*

The recurring telephone calls and *„the possibility to ask questions “ (Participant 7, GP)* made patients feel more comfortable in coping with COVID-19 and helped to reduce their fear which was most prominently underscored as positive outcome:

“*We noticed that they got sick and they are at home, they just sit there (…) (that is why) they found it (the recurring telephone calls) helpful, because they were contacted again and again.” (Participant 10, VERAH)*.

Consequently, HCP reported that patients perceived the management with CovidCare as better quality of care because *“these follow-up calls ‘how are you, is everything okay, are you symptom-free?’, that is indeed not a matter of course.“ (Participant 4, GP).* However, one interviewee considered CovidCare as too extensive arguing that some patients with severe symptoms do not want to be bothered being asked many questions.

### Adoption

Within adoption we assessed the characteristics of participating practices as well as the reasons for and intention to adopt CovidCare in general practices, namely facilitators and barriers.

Considering the characteristics of the participating HCP, they mostly employed one or two VERAHs. The latter contributed to an easier integration into daily practice as two VERAHs either shared their responsibility for CovidCare or one VERAH managed the CovidCare patients and the other was responsible for other tasks. Otherwise, the workload was perceived as high for only one VERAH:

*“I only have one VERAH in the practice and she can’t do everything alone. She makes home visits and also other things and she is also responsible for CovidCare.” (Participant 6, GP)*

Besides, most HCP had previous experience with other modules within the CareCockpit software. They considered this as helpful for the adoption of CovidCare because they were already familiar with the software and how and when to use the intake, assessment an monitorings:

*“When you have been doing this [using the CareCockpit software] for a long time, then this [CovidCare] is very easy to do.” (Participant 10, VERAH)*

The interviewees expected to gain more knowledge and to collect data in a structured way through CovidCare. They were interested in getting to know *“more about the disease and the course of the disease” (Participant 4, GP)* especially due to a lack of evidence-based research and official guidelines and consequently a particularly high level of uncertainty. Hence, as another main reason for using CovidCare, HCP expected CovidCare to facilitate the *“provision of the best possible care” (Participant 11, VERAH; Participant 10, VERAH*) for COVID-19 patients. Furthermore, financial remuneration was less commonly mentioned and therefore seem to play a rather less prominent role compared to the more intrinsic motivational aspects for the adoption of CovidCare.

Overall, an easy, time-saving integration into daily practice routines was underscored as crucial prerequisite for the adoption of CovidCare. This also included the questions to be as short and as necessary as possible. Therefore, HCP who were already familiar with the CareCockpit software had a more positive attitude on CovidCare whereas VERAHs who did not use the software before were more reluctant. Before using CovidCare, they were concerned as to whether CovidCare would be too time-consuming, e.g. regarding the documentation effort, while maintaining the daily operation of the practice:

*“What does it cost me in terms of time, when I can’t do other things or I have to do everything anyway, I have to add it on and that costs me even more time, I can’t just say ‘no home visits will be scheduled for next week’ in order to manage CovidCare.” (Participant 11, VERAH)*

As a result, timely and personnel resources were identified as major barriers for the adoption of CovidCare by most HCP. Some HCP also described the process of familiarisation with CovidCare as difficult and time-consuming. Therefore, providing technical support for the installation and initial operation of CovidCare was required as necessary. Doubts regarding patients’ acceptance were less common.

### Implementation

Implementation refers to the fidelity of CovidCare, how CovidCare has been delivered, the adaptions that were necessary, including by whom and why, as well as problems and cost of implementing CovidCare in daily practice. Implementation served as key dimension for the evaluation of CovidCare in order to tailor it to the requirements of the HCP and foster an easy use and integration into their daily practice.

Participating HCP used CovidCare differently. While some HCP used it as intended (e.g. specific/shared responsibilities for GPs and VERAHs respectively, three to four recommended contacts with the patients), others reported that the VERAH was mainly responsible for all aspects of CovidCare. In these cases, the GP performed a controlling function:

*”When I knew the patients well, [then] I asked about the chronic diseases and I presented it to the GP and he checked whether he considered anything else important in addition or not.” (Participant 10, VERAH)*

It turns out that VERAHs play a key role in the CovidCare management, which might also be linked to the GPs’ expectation that CovidCare can be a relief of their resources. Moreover, the use of CovidCare varied from complete to partial adoption. Against the background of limited time and personnel resources, HCP stated that adaptions were necessary. Specifically, some HCP, mostly VERAHs, were overwhelmed with too many questions or the number of recurring telephone monitorings due to their limited resources in the pandemic. For some patients, specific components of CovidCare were left out, illustrated by the following quote:

*“So, for one patient we couldn’t give him a pulse oximeter to take home (…) there was no-one who could have gotten it from our practice. And then we just skipped things like that because it wasn’t technically feasible.” (Participant 18, GP)*

However, reducing the number of questions or the number of monitorings were identified to mitigate this barrier. Hence, they expected a shorter version of CovidCare to be more feasible. To account for this, we released a shorter version of CovidCare to make CovidCare better meet the HCP’s requirements.

Regarding the problems, time and cost of CovidCare, the time factor has been identified as major problem, including the time for registration, software installation and initial operation, *“especially in the peak phase of the pandemic (…) there was almost no time for it.” (Participant 16, VERAH).* This also contributed to a higher workload for VERAHs. Therefore, VERAHs reported that having their schedules blocked during CovidCare sessions was crucial for getting familiar with it and the integration into daily practice.

Furthermore, interviewees recognised problems on the software level. For some, the inclusion criteria were not clearly enough presented in the software, others stated that they were only able to include a few patients who met the inclusion criteria such as being insured with a specific sickness fund. Moreover, some interviewees disliked that every HCP had to create an own account in order to individually use CovidCare as VERAH or GP respectively. Due to the time constraints, an easier handling of the software (e.g. less questions, installation by the software provider) might have contributed to an easier use and faster integration into daily practice. Nevertheless, some HCP underscored that CovidCare was easy to integrate. They attributed this to their previous experience with the CareCockpit software or a second VERAH.

### Maintenance

Maintenance describes the extent to which CovidCare became part of the daily practice routines of GP’s practice. It also includes suggestions for adaptations to improve the fit between CovidCare and general practices in order to enable CovidCare to become part of organizational practice.

Most interviewees stated that they are planning to continue the use of CovidCare. They pointed out the importance of being able to provide structured care adding that CovidCare offered an orientation to keep track on the course of high-risk COVID-19 patients. Moreover, from the HCP’s perspective, patients valued the management with CovidCare as positive and were thankful for the additional time spend during the recurring phone calls:

*“We have heard from many patients that they really liked that they were taken care of, and that’s why we thought it’s a good idea and we’ll continue to do it [CovidCare].” (Participant 17, VERAH)*

Nevertheless, some HCP were not sure whether they will use CovidCare in the future:

*“To be honest, I don’t know yet. So, I would like to [use CovidCare], if you could offer a shorter version (…), a few questions will be enough.” (Participant 19, GP)*

They considered the components of CovidCare as too extensive and as an additional burden during the COVID-19 pandemic and the already increased workload.

In order to improve CovidCare and facilitate an easy integration into daily practice routines, HCP made several suggestions for improvement. First and foremost, they considered less questions as particularly helpful to save time and make CovidCare less burdensome for HCP in general and VERAHs in particular. Second, they suggested to expand the inclusion criteria and include all patients with COVID-19, e.g. regardless of which sickness found they are insured with. By including all COVID-19 patients, they would be able to conduct more assessments and monitorings and therefore get familiar with the software more easily. Third, they mentioned several other aspects for improving CovidCare such as providing an app (for patients to document their vital signs), additional questions (e.g. regarding the patients’ occupation in order to detect how patients got infected), including an email reminder (which reminds the VERAH to conduct the monitoring at the scheduled time) and the integration of the CovidCare data into the practice management software.

## Discussion

The aim of this qualitative process evaluation study was the evaluation of HCP’s utilisation of CovidCare, a software tool for the structured surveillance of high-risk home-quarantined COVID-19 patients in general practice. The interviews with GPs and VERAHs who used CovidCare and participated in the CovidCare study gave valuable insights and feedback regarding the dimensions of the RE-AIM framework. Before using CovidCare, HCP managed COVID-19 patients differently, however mostly rather unstructured.

Overall, as for the ‘reach’ dimension of the RE-AIM framework, HCP stated that the patients’ willingness to be managed within the remote CovidCare was high, especially in older patients. But HCP also treated patients within CovidCare who did not meet all inclusion criteria in order to provide adequate health care for all COVID-19 patients. Considering the ‘effectiveness’ of CovidCare, HCP attributed their overall high satisfaction with the module to 1) the orientation of CovidCare due to missing body of evidence-based guidelines 2) the possibility to gain new knowledge about the patients and the disease and 3) a structured data collection using CovidCare. GPs also highlighted a relief of workload, as in most cases the VERAH conducted large parts of the CovidCare management. In contrast, some VERAHs perceived an additional workload as particularly negative against the background of an already increased workload due to the pandemic. However, VERAHS emphasized that they enjoyed the CovidCare management as most patients provided particularly positive feedback and felt less anxious about coping with their disease. Previous experience with the software, an easy integration into daily practice as well as the possibility of a structured data collection were considered as major facilitators for the ‘adoption’ of CovidCare. Subsequently, time and personnel resources were identified as major barriers. Adaptions such as skipping questions were necessary to facilitate the ‘implementation’ of CovidCare. As for the ‘maintenance’ dimension, most HCP stated that they will continue to use CovidCare, advocating for less-time consuming version of CovidCare or an app for the patients.

The COVID-19 pandemic shortly led to an overwhelmed health care system worldwide. General practices play a significant role in the management of COVID-19 patients, as they are responsible for assessing ambulatory patients, also in order to prevent hospitals from being overwhelmed. However, most countries did not have emergency plans that clearly stated how general practices could be supported in a sustainable way which caused uncertainty among health care providers [23, 24]. As recommended by the World Health Organisation, remote monitorings which provide guidance on the management of COVID-19 can be a viable means for the treatment of the group of infectious COVID-19 patients and can support general practices in health care delivery [3–5, 25]. In order to provide the best possible care, it is necessary to evaluate the perspective of patients and providers on remote monitorings and supporting tools.

Most existing telemedicine models for the management of COVID-19 patients only include an unilateral contact to HCP from the patients’ initiative [10]. However, this might affect an early detection of worsening symptoms and can therefore lead to a less effective management of COVID-19 patients. Within CovidCare, HCP and patients stay in (close) contact, depending on the individual medical condition, symptoms and risk factors of the patient, which was underscored by the interviewees as particularly helpful.

A study on the patients’ use of an app to support them with COVID-19 education, self-assessment and monitoring of their health status without continuous contact to a HCP found that users discontinued to use the symptom diary. The authors attributed this to the generic messages and therefore a lack of personal feedback that needs to be individually tailored to the patient [9]. Our study adds that, interviewees perceived a software tool (CovidCare) as valuable due to the standardized symptom diaries that can be individually tailored to the patient including individual feedback can be given to the patients in the telephone monitorings, including the opportunity for patients to ask questions. Furthermore, telephone monitorings enable a structured surveillance of patients, of whether the patient uses the symptom diary.

This helped HCP and patients to reduce uncertainties which was underscored as most prominent positive effect. Individually tailored and close feedback and monitorings were also reflected as helpful by previous studies [26, 27].

Moreover, another study, as well as our results, points to the importance of a direct patient-HCP-contact, e.g. via telephone calls and underscores that self-monitoring is somewhat limited as patients need guidance [10]. Our study adds, that besides the patients’ benefit, CovidCare also provided guidance for HCP as it helped them to keep track of their patients and which symptoms and vital parameters to focus on.

Another study on remote patient monitoring of infectious patients in ambulatory care found that participating practices identified the integration of the monitoring tools as a main obstacle and could not benefit from a lower workload, which is somewhat in line with our findings on perceived barriers [8]. However, we explored that CovidCare led to a shift of resources: while VERAHs perceived additional workload as negative, GPs highlighted a relief of their resources as positively impacting the utilisation of CovidCare. Notwithstanding, VERAHs emphasized that they enjoyed the CovidCare management due to the patients’ positive feedback and previous experience with the software facilitated the familiarization with it. Therefore, we consider the integration of software tools into existing practice software as mandatory for a sustainable implementation and use in daily practices.

Considering other prerequisites that enable an easy integration into daily practice and therefore the utilisation of a tool, previous research underscored that it is crucial that monitoring and surveillance data can be integrated into the existing practice management software [7, 8, 12], which is in line with our findings.

A study on an app to provide HCP with a software to remotely monitor COVID-19 patients in home-quarantine found that the app reduced paperwork for HCP because patients self-assessed their health status [11]. Still, HCP spend additional time on assisting patients in activating and using the app. HCP in our study also mentioned an app for patients’ self-documentation to further improve the tool and potentially save time for HCP. Moreover, as some HCP in our study perceived the process of familiarisation with CovidCare as time-consuming, our study adds that providing technical support for the installation and initial operation of a tool for HCP and patients by the software provider is required as a crucial prerequisite, especially against the background of a relief of resources. As suggested by some of the interviewees, especially for patients with mild symptoms, self-assessment via a self-monitoring app can be of value for both patients to facilitate their active participation and for HCP to reduce workload [28]. We used our results to further improve CovidCare and to adapt it to meet the requirements of the general practices. For instance, we a) cut some questions which were not absolutely necessary for the assessment of patients’ conditions such as a patient decree, COVID-19-independent vaccination status (e.g. influenza), b) adapted the inclusion criteria according to the current RKI recommendations, and c) simplified the presentation of questions and response options. Based on the ongoing telephone support we provide for participating practices, we will further improve the tool and continuously tailor it to the HCP’s needs.

Furthermore, for the nationwide implementation of CovidCare it would be necessary to include it in the service catalogue of the Association of Statutory Health Insurance Physicians. Moreover, to reduce HCPs workload and encourage patients’ self-management, we are currently developing an app which can be used as digital symptom diary. In addition, financial remuneration for HCP seems to be important to reward their performance, to acknowledge their enormous workload and support continuous care for COVID-19 patients as well as non-COVID-19 patients. As previous experience with the software tool seems to facilitate utilisation, future research should investigate how existing models can be supplemented by integrating support tools for the management for example of highly prevalent diseases in order to foster an easy implementation into daily practices.

### Strengths and limitations

Due to the purposeful sampling strategy, the sample might be biased as perhaps HCP with a rather positive view on CovidCare agreed to participate in an interview. Although 27 HCP showed interest in participating in our study, we realised interviews with seven GPs and 12 VERAHs because eight persons have not yet used CovidCare or did not yet have any eligible patient to include into CovidCare. Furthermore, we can make no statements of the reasons for refusing to use CovidCare because only HCP who used CovidCare participated in the interviews. To account for this, we explicitly encouraged all participants to reflect on barriers and problems regarding the utilisation of CovidCare and make suggestions for improvement. Thus, we were able to elicit a broad range of perspectives that included positive and negative aspects of the utilisation of CovidCare. As recommended by Glasgow et al., apart from employing all RE-AIM dimension to the data, we especially aimed to understand how HCP used CovidCare and why they used it the way they did [29]. Therefore, we encouraged the participants to reflect on the daily utilisation of CovidCare and adaptions that were necessary. The RE-AIM framework proofed to be viable to guide the study. Specifically, we developed the interview guide based on the RE-AIM dimensions and used these to analyse the data as well as for reporting. Hence, were able to follow a coherent approach for our study to ensure transparency and comprehensibility. Challenges of sometimes difficult specific attribution of statements to a RE-AIM dimension, e.g., barriers to CovidCare adoption and implementation issues, were addressed by having two experienced researchers from different disciplines independently code the transcripts and continuously exchanged on the data and interpretations. This allowed us to sufficiently analyse all the data and produce consistent findings.

We did not invite the interviewees to provide feedback on our findings through member checking which can be used for the purpose of data validation. Due to the already high workload of the participants due to the pandemic, the purpose was to keep the additional effort for the interviews to a minimum.

## Conclusion

The COVID-19 surveillance and care tool for COVID-19 patients with increased risk was perceived as useful by GPs and VERAHs as it facilitated the identification of disease deterioration and provided a guidance on the management of high-risk COVID-19 patients in home-quarantine. Subsequently, it reduced uncertainties for both patients and HCP who can also benefit from a relief of their workload.

As major benefits, software tools like CovidCare can be an orientation for HCP and patients alike and an opportunity to earn knowledge and gather structured data especially in a new and dynamic setting such as the COVID-19 pandemic. To maintain comprehensive and continuous health care during a pandemic, it is useful to employ supportive remote health care supplies. This qualitative study gave valuable feedback for the further development of the tool. Against the background of limited resources in general practices in dynamic times of the pandemic, these aids should be as simple as possible yet as comprehensive as necessary.

## Supplementary Information

**Additional file 1.**

**Additional file 2.**

**Additional file 3.**

## Data Availability

The datasets generated and analysed during the current study are not publicly available due to privacy reasons of the individual participant but are available from the corresponding author on reasonable request.

## Abbreviations

VERAH: Care Assistant in General Practice with special training, Versorgungsassistentin in der Hausarztpraxis
CovidCare: COVID-19 surveillance and care tool for COVID-19 patients with increased risk
GPs: General practitioners
GP-centred care: General-practice centred care
HCP: Health care providers
