# Supplementary material for: Health care professionals’ perspectives on the utilisation of a remote surveillance and care tool for patients with COVID-19 in general practice: a qualitative study
Source: BMC Prim Care. 2022 Sep 27;23:254. doi: 10.1186/s12875-022-01863-z (PMC9513296; doi:10.1186/s12875-022-01863-z)
Supplement: Supplementary file 1 — Additional file 1. [file 12875_2022_1863_MOESM1_ESM.docx]

**Process evaluation:**

**Surveillance and care for confirmed and suspected patients with COVID-19**

**in general practice (CovidCare)**

**Interview guide for general practitioners and VERAHs**

(finalised version as of 31^st^ January 2021)

*1. Reach*

- What motivated you to use the CovidCare-module and participate in the CovidCare study?
- Only for general practitioners: How did you decide whether or not a patient will be monitored within the CovidCare-module/will be invited to participate in the CovidCare study?
  - How were patients made aware of the study?
  - Were patients deliberately not made aware of the study?
- How would you describe the willingness of patients to be treated in the CovidCare-module and participate in the CovidCare study?
- How would you describe the patients who have been treated within the CovidCare-module?

*2. Efficacy*

- How satisfied were you with the use of the CovidCare-module?
- Which aspects of the CovidCare-module have you used?
- Which aspects did you find (not) helpful and why?
- To what extent has the use of the CovidCare-module influenced patient care? Do you consider this as helpful, why?
  - Which effects did you recognize for patients? Do you consider this as helpful, why?
- To what extent has the use of the CovidCare-module influences your work (positively and negaltively)?

*3. Adoption*

- Why did you decide to implement the CovidCare-module?
  - Which expectations and concerns did you have?
  - What has (not) been fulfilled?
- What advantages and disadvantages do you anticipate in using the CovidCare-module?
  - - Why did some general practitioners and/or VERAHs in your practice use the CovidCare-module and others not?

*4. Implementation*

- - - How do you use the CovidCare-module? Can you describe the process with an example of a patient?
  - Who conducted the assessment, monitoring etc.?
    - To what extent have you made adjustments? (e.g. regarding the number of assessments, monitorings and the questions)
    - How did you treat Covid-19-patients before using the CovidCare-module?

*5. Maintenance*

- - - Will you continue to use the CovidCare-module in your practice? Why (not)?
    - How do you assess the chances of a widespread implementation of the CovidCare-module?
  - Which facilitators and barriers do you anticipate?
- What would you suggest to facilitate the usage of the CovidCare-module?
- Which importance do you attribute to app-based applications?

*6. Interview termination*

- Are there any remaining aspects important to you that we have not addresses so far?
- Do you have any further questions?
