# Supplementary material for: Health care professionals’ perspectives on the utilisation of a remote surveillance and care tool for patients with COVID-19 in general practice: a qualitative study
Source: BMC Prim Care. 2022 Sep 27;23:254. doi: 10.1186/s12875-022-01863-z (PMC9513296; doi:10.1186/s12875-022-01863-z)
Supplement: Supplementary file 3 — Additional file 3. [file 12875_2022_1863_MOESM3_ESM.docx]

**Additional file 3/Table 2: Summary of key dimensions and subdomains**

| *Key dimension* | *Definition* | *Subdomains* | *Supporting quotes* |
| --- | --- | --- | --- |
| Reach | Reach describes the extent to which patients are reached by the implementation of CovidCare and refers to patients who were willing or refused to be managed with CovidCare and why | Patients’ characteristics | *“many patients with hypertension and two or three with asthma” (Participant 14, VERAH)* |
|  |  | Patients’ willingness/refusal | *“It actually went down quite well except for one person (…) but most were actually quite grateful that we took care of them.”*  *(Participant 14, VERAH)* |
| Effectiveness | Effectiveness is defined as positive and negative outcomes for patients and HCP, the patients’ quality of life as well as the patients and the HCP’s satisfaction with CovidCare | *Patients’ satisfaction* | *„They were definitely happy to be taken care of and some of them cried because they felt alone without any contact to a GP or the public health department for days and at the end the were very, very grateful.” (Participant 1, VERAH)* |
|  |  | Effects on patients | *“I found that the patients felt much better cared for, because we had regular contact with the patients and they knew, that someone was there to take care of them and that the communication between the GP and the VERAH and the patient worked out well.”*  *(Participant 9, VERAH)* |
|  |  | HCP’s satisfaction | *“So basically, I had exactly this expectation that a structured documentation and monitoring or support of patients is possible and CovidCare actually met my expectations.”*  *(Participant 18, GP)* |
|  |  | Effects on HCP | *Question: “What did you find particularly helpful (…)?”*  *Answer: It served as a guideline (…) simply that I already have a guideline on (what to ask, for example) cough, cold, hoarseness, diarrhea.”*  *(Participant 14, VERAH)* |
| Adoption | Adoption refers to the characteristics of participating practices as well as the reasons for and intention to adopt CovidCare in general practices, namely facilitators and barriers. | Characteristics of participating practices | *“We had a practice takeover from older to younger GPs and they are obviously already open to everything new [e.g. CovidCare].”*  *(Participant 10, VERAH)* |
|  |  | Reasons for participating | *“In the beginning I also found it interesting (…) to document [symptoms] and see (…) the difficulties they had during domestic isolation, to see and empathise, (…) because in the beginning we simply didn’t know anything about Covid.”*  *(Participant 15, VERAH)* |
|  |  | Facilitators | *“Expectations about this CovidCare-module… the were more statistical and to get a better orientation in this field.”*  *(Participant 6, GP)* |
|  |  | *Barriers* | *“[I had] relatively few concerns, there were more practical concerns; will this cost too much effort, including documentation effort and time exposure.”*  *(Participant 13, GP)* |
| *Key domains* | *Definition* | *Subdomains* | *Supporting quotes* |
| Implementation | Implementation is defined as the fidelity of CovidCare, how CovidCare has been delivered, the adaptions that were necessary, including by whom and why, as well as problems and cost of implementing CovidCare in daily practice. | Delivery of CovidCare | *“So yes, we [VERAH and GP] work together. I actually know all the steps of the patients, the complete course of the disease. And we do that together: what and how to fill out [the module].”*  *(Participant 6, GP)* |
|  |  | Adaptions | *“I didn’t ask some of the questions. As I said, I usually let the patient tell me about how he was doing and the asked more specific questions, but I didn’t as everything.”*  *(Participant 14, VERAH)* |
|  |  | Problems/time/cost | *“Well, I think it’s too time-consuming. Or too complex. At least that’s how we perceived it.”*  *(Participant 4, GP)* |
| Maintenance | Maintenance describes the extent to which CovidCare became part of the daily practice routines of GP’s practice, including suggestions improvements. | Intention to continue to use CovidCare | *“I have to say, if it was simplified, I would probably use it a lot more and if it were possible to include more patients, not only for AOK patients.”*  *(Participant 16, VERAH)* |
|  |  | Suggestions for improvements | *“A shorter version. That would be my only suggestion for improvement.”*  *(Participant 9, VERAH)* |
